# Predictors of Premature Mortality Following Coronary Artery Bypass Grafting: An Iranian Single-Centre Study

**DOI:** 10.3390/healthcare12010036

**Published:** 2023-12-23

**Authors:** Fatemeh Pakrad, Rahman Shiri, Azadeh Mozayani Monfared, Ramesh Mohammadi Saleh, Jalal Poorolajal

**Affiliations:** 1 Chronic Diseases (Home Care) Research Center, Hamadan University of Medical Sciences, Hamadan 6517838698, Iran; f_pakrad@yahoo.com; 2 Finnish Institute of Occupational Health, 00032 Helsinki, Finland; rahman.shiri@ttl.fi; 3 Department of Cardiology, School of Medicine, Hamadan University of Medical Sciences, Hamadan 6517838736, Iran; azadeh_m27@yahoo.com; 4 Department of Nursing, School of Nursing and Midwifery, Hamadan University of Medical Sciences, Hamadan 6517838698, Iran; r.mohammadisaleh@umsha.ac.ir; 5 Department of Epidemiology, School of Public Health, Hamadan University of Medical Sciences, Hamadan 6517838687, Iran; 6 Modeling of Noncommunicable Diseases Research Center, School of Public Health, Hamadan University of Medical Sciences, Hamadan 6517838687, Iran

**Keywords:** cohort study, coronary artery bypass grafting, glomerular filtration rate, obesity, risk factors

## Abstract

Modifiable risk factors play an important role in the premature mortality among patients undergoing coronary artery bypass grafting (CABG). The aim of this study was to examine the factors that influence the early death of patients who had CABG. We conducted a prospective cohort study and followed 2863 patients after their CABG, and collected data on their characteristics and blood tests. We used the Cox proportional hazards regression model in Stata, version 16, to identify the predictors of early mortality. Out of 2863 patients, 162 died during the follow-up period. The survival rate was 99.2% within the first three days after the surgery, 96.2% from the fourth day to the end of the first year, 94.9% at the end of the second year, and 93.6% at the end of the third year. After adjusting for confounding factors, we found that older age (hazard ratio [HR] 1.05, 95% CI 1.02, 1.08 for one year increase in age), obesity (HR 2.16, 95% CI 1.25, 3.72), ejection fraction < 50% (HR 1.61, 95% CI 1.06, 2.44), number of rehospitalizations (HR 2.63, 95% CI 1.35, 5.12 for two or more readmissions), history of stroke (HR 2.91, 95% CI 1.63, 5.21), living in rural areas (HR 1.58, 95% CI 1.06, 2.34), opium use (HR 2.08, 95% CI 1.40, 3.09), and impaired glomerular filtration rate increased the risk of early death after CABG, while taking a beta-blocker (HR 0.59, 95% CI 0.38, 0.91) reduced the risk. We conclude that modifiable risk factors such as excess body mass, high blood glucose, opium use, and kidney dysfunction should be monitored and managed in patients who had CABG to improve their survival outcomes.

## 1. Introduction

Patients with coronary artery disease often undergo coronary artery bypass grafting (CABG), a surgical procedure that restores blood flow to the heart. In the United States, about 500,000 CABG surgeries are performed every year [1]. However, CABG is not without risks, and can lead to postoperative complications [2,3]. Postoperative complications are a major cause of readmission after CABG, despite the improvements in surgical techniques [2]. According to previous research, 14% of patients who had CABG experienced complications, and 2% died within 30 days after the surgery [4]. Another study found that the increased risk of death and rehospitalization persisted for up to 7 years after CABG [5]. The mortality rate of CABG patients at 1 year after the surgery is around 5% [6]. According to research in Iran, a study on all kinds of heart surgeries revealed that isolated CABG was the most common procedure with the lowest in-hospital mortality rate of 0.5% [7]. The percentage of patients who survived one year and five years after CABG was 92% and 83%, respectively [8]. Another Iranian study reported that the survival rate of patients who underwent CABG with coronary endarterectomy was about 80% after 20 months of follow-up [9].

Previous research has shown that black patients have a higher mortality rate than white patients after CABG [10], and that socioeconomic factors such as low education, low income, and being single [11] are also linked to higher mortality. Smoking history [12] is another factor that increases the risk of death from any cause in patients with coronary artery disease who undergo CABG. On the other hand, some studies have reported lower mortality and fewer adverse cardiovascular events and hospital readmissions after CABG in overweight patients [13,14,15]. Postoperative complications such as atrial fibrillation [16] and acute kidney injury [17] are associated with higher short-term and long-term mortality after CABG, as well as longer stays in the intensive care unit and hospital [2]. However, few studies have examined the modifiable risk factors for early death after CABG in Iranian patients with coronary artery disease [18,19,20], and none have focused on long-term outcomes. Understanding the causes of early death after CABG is crucial for developing better treatment and prevention strategies to improve survival rates [21]. The purpose of this study was to identify the factors that influence early death after CABG in Iranian patients with coronary artery disease.

## 2. Subjects and Methods

We conducted a cohort study of CABG patients at Farshchian Heart Centre, the main specialized cardiovascular centre in western Iran. We retrospectively reviewed the medical records of eligible patients who underwent CABG from September 2015 to October 2017, and prospectively followed them up from October 2017 to March 2020. We collected the baseline characteristics from the medical records and the survival and mortality data from phone calls with patients or their families. We included patients who underwent CABG between October 2015 and March 2020 (N = 3156 patients). We excluded patients who had other cardiac surgeries such as heart valve replacement or repair, atrial and ventricular wall repair, or cardiac tumour resection (N = 293 patients). Patients who had typical chest pain or positive noninvasive tests for ischemia underwent angiography. Patients who had one-, two-, or three-vessel disease or left main coronary artery disease and were not suitable for angioplasty according to the guidelines underwent CABG. The final sample size was 2863 patients. In this study, we obtained the patients’ data from their medical records, and followed-up with them once by phone. For 638 patients, the follow-up ended until the patients’ discharge from the hospital, and we were not able to contact them further. The remaining patients were contacted at various time points after surgery, ranging from a few days to five years.

We collected data on demographic, behavioural, and clinical variables of the patients, such as age, sex, weight, height, residence area, smoking status, opium use, hypertension, diabetes, previous angioplasty or surgery, recent myocardial infarction, number of bypassed vessels, atrial fibrillation before and after surgery, beta-blocker use, stroke history, cardiopulmonary bypass (on-pump or off-pump), pulmonary diseases, and readmission. The sources of the data were the patient’s health record, medical history, clinical report, nursing notes, sonography report, angiogram, and surgical report. We also measured the blood levels of various biomarkers, including fasting blood glucose, white blood cells, neutrophil to lymphocyte ratio, red blood cells, haemoglobin, haematocrit, total cholesterol, triglycerides, LDL and HDL cholesterol, blood urea nitrogen (BUN), creatinine, glomerular filtration rate (GFR) and cardiac ejection fraction. We defined smoking as current smoking. We calculated body mass index (BMI) as weight divided by height squared (kg/m^2^) and categorized it as normal (BMI < 25 kg/m^2^), overweight (BMI 25–29.9 kg/m^2^), or obese (BMI ≥ 30 kg/m^2^). We defined normal blood glucose as fasting blood glucose level below 99 mg/dL, prediabetes as fasting blood glucose level between 100 and 125 mg/dL, and diabetes as fasting blood glucose level above 126 mg/dL. The outcome of interest was death from any cause within the five years after CABG.

We used the Sysmex kx-21 N device, which employs electrical resistance technology, to perform blood cell counts. We defined white blood cell counts of 10,000/mm^3^ or more as elevated. We defined low levels of red blood cells, haemoglobin, and haematocrit as less than 3.9 mL/m^3^, less than 11.5 g/dL, and less than 36%, respectively. A pathologist verified the neutrophil to lymphocyte ratio obtained from the device and confirmed it. We considered a ratio of 2.15 or higher as an elevated neutrophil to lymphocyte ratio [22,23]. We defined serum cholesterol levels of 200 to 239 mg/dL as borderline, and above 240 mg/dL as high. We defined triglyceride levels of 200 to 399 mg/dL as borderline, and above 400 mg/dL as high. We defined HDL levels below 30 mg/dL as low, and LDL levels above 150 mg/dL as high. We assessed kidney function with BUN, creatinine, and GFR. We defined high BUN and blood creatinine levels as above 25 mg/dL, above 1.1 in women, and above 1.4 mg/dL in men, respectively. We classified GFR into six categories: (1) normal (>90 mL/min/1.73 m^2^), (2) slight decrease (60 to 89), (3) mild to moderate decrease (45 to 59), (4) moderate to severe decrease (30 to 44), (5) severe reduction (15 to 29), and (6) renal failure (<15 mL/min/1.73 m^2^) [24]. We classified fasting blood glucose levels into three levels: normal (70 to 99 mg/dL), borderline (100 to 125 mg/dL), and diabetic (> 126 mg/dL) [25]. We measured all of the above biochemical tests using a BT 3500 device (Biotecnica Instruments S.p.A, Rome, Italy).

### Statistical Analysis

We applied univariate and multivariate Cox proportional hazards regression models to analyze this time-to-event data. We first performed a univariable Cox proportional hazards regression model for each variable, and then included all the variables with *p*-value < 0.30 in the univariable models in the multivariable models that estimated premature mortality. The variables with *p*-value ≥ 0.05 were removed from the final multivariable model. We used Stata, version 16 for the analyses.

## 3. Results

The final analysis included 2863 patients. The mean age of the patients was 62.9 ± 9.4 years, and the majority of the patients were in the 60–69 years old age group (40.17%, N = 1150). Men accounted for 73.38% (N = 2101) of the surgical cases. Most of the patients resided in urban areas (76.39%, N = 2187). The prevalence of hypertension was 49.95% (N = 1430), and 32.06% of the patients were on beta-blocker therapy (Table 1).

**Figure 1:**
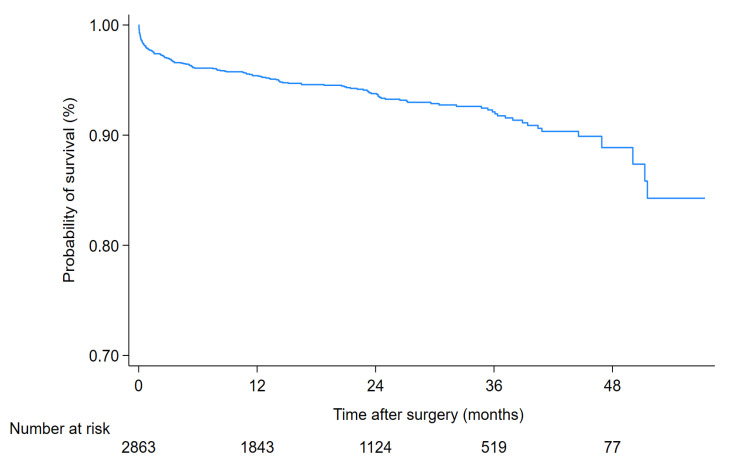
Survival rate of the 2863 patients who underwent CABG.

Out of 2863 patients who underwent CABG, 162 died within five years. The survival rate after CABG was 99.2% within the first three days after the surgery, 96.2% at the end of the first year, 94.9% at the end of the second year, and 93.6% at the end of the third year (Figure 1 and Table 2).

Cardiovascular causes accounted for most of the deaths (126 cases, 77.8%), while the rest (36 cases, 22.2%) were due to noncardiac causes. The noncardiac causes were stroke (*n* = 13), cancer (*n* = 10), COVID-19 (*n* = 5), COPD (*n* = 2), diabetes (*n* = 2), pelvic fracture (*n* = 2), and kidney failure (*n* = 2) (Table 3).

As shown in Table 4, age, place of residence, opium use, ejection fraction, history of stroke, beta-blocker use, and the number of rehospitalizations were associated with mortality after CABG. There was no significant difference in the all-cause mortality rate between men and women.

Table 5 shows the univariable hazard ratios of how laboratory characteristics relate to mortality rate after CABG.

The associations of clinical and paraclinical characteristics with all-cause mortality rate after CABG are shown in Table 6, using multivariable hazard ratio (HR) analysis. Age was a significant predictor, and the mortality risk increased with age (HR 1.05, 95% CI 1.02, 1.08 for one year increase). Obese patients had a 2.16-fold higher mortality rate after CABG. The number of postsurgery hospitalizations was also an important factor, with a 2.63-fold (95% CI 1.35, 5.12) higher mortality risk for those who were hospitalized two or more times. The mortality rate after surgery increased with the decrease in ejection fraction (HR 1.61, 95% CI 1.06, 2.44). A lower glomerular filtration rate (GFR) was associated with a higher risk of death. Patients with GFR less than 29 had an 8.68-fold higher risk of death after coronary artery bypass surgery. Moreover, history of stroke, opium use, prediabetes, and living in rural areas were associated with increased risk of death after CABG. Beta-blockers had a protective effect after CABG (HR 0.59, 95% CI 0.38, 0.91).

## 4. Discussion

The current study indicates that several factors, such as obesity, atrial fibrillation, reduced ejection fraction, high blood glucose, and low glomerular filtration rate increase the risk of death from any cause after CABG in patients with coronary disease.

The role of sex in short- and long-term survival after CABG is unclear. Some studies have reported longer survival for women than for men after CABG [26], while others have found the opposite [27]. Moreover, one study [21] reported higher mortality for women within 1 year after CABG, but higher mortality for men between 1 and 7 years after CABG. However, in the current study, we did not observe any sex difference in mortality rate. The mean age of patients was high for both sexes, and the present study showed that the most important predictor of mortality after CABG is the patient’s age rather than patient’s sex. This is consistent with another study in Iran that found no sex difference in postoperative mortality [20]. As the prevalence of comorbid chronic conditions rises among the elderly population, they need to have improved perioperative care procedures [28].

Our study revealed that preoperative renal disease is a significant predictor of mortality after CABG. This is consistent with a previous study that reported a higher mortality rate among patients with chronic renal failure 1 to 7 years after CABG [21]. Obesity is a well-known risk factor for cardiovascular disease. However, some studies have suggested a paradox of obesity, where overweight and obese patients with cardiovascular disease have a better outcome after CABG than normal-weight patients with cardiovascular disease [29]. A meta-analysis [30] found that overweight patients, but not obese patients, had lower short-term and long-term mortality after CABG than normal-weight patients. Another meta-analysis [31] showed that mortality after cardiac surgery was not affected by a slight increase in body mass index, but was worse for underweight and extremely obese patients. According to a study conducted on Iranian patients (N = 235), there was no difference in the mortality rates at the hospital or after 3 months of CABG between obese and nonobese patients [32]. However, that study compared patients having a BMI of 30 kg/m^2^ or above with patients who had a BMI below 30. The inclusion of overweight patients in the comparison group might have weakened the relationship. In contrast to these findings, we observed that obesity was associated with a 1.79-fold higher mortality rate than normal weight after CABG, while there was no difference between normal-weight and overweight patients.

Diabetes and hypertension are common comorbidities in patients with chronic renal disease and cardiovascular diseases. Diabetes [33] and arrhythmias, especially atrial fibrillation [16,34], are prevalent in patients with coronary disease who undergo CABG. In agreement with a prior study [35], we found that prediabetes was associated with a higher mortality rate than nondiabetes after CABG. This could be explained by the inadequate management of blood glucose levels in patients with borderline blood glucose.

Our study showed that beta-blockers could lower the mortality rate after CABG. However, the effect of beta-blockers on mortality and major cardiac complications in patients with coronary heart disease remains unclear. Some evidence suggests that beta-blockers do not affect major cardiovascular events in patients with coronary artery disease [36], while other studies indicate that beta-blockers could decrease the mortality rate in patients who had a recent heart attack [37] or heart failure with systolic dysfunction [38]. In addition, the use of beta-blockers in patients with recent myocardial infarction was linked to a lower risk of a heart attack [39].

We found that rural residents had a higher mortality rate after CABG than urban residents. This may be because rural residents have more risk factors for heart disease and more severe heart problems due to less access to healthcare services. Another possible factor that may account for the increased risk of death after CABG among people living in rural areas compared to those living in urban areas is the lower educational level of the former group [40]. Moreover, in people with low education and cardiometabolic comorbidities, there may be an effect on the progression of chronic diseases. It is important to improve the preventive strategies for this specific patient group [41]. Education is a key factor that influences the overall heart risk in people with high blood pressure. Therefore, it should be carefully evaluated and included in the management of high blood pressure and heart risks [42]. Smoking did not predict the mortality rate in our study. The lack of association was due to comparing current smokers with never or past smokers. Past smokers quit smoking because of their heart disease. Our study showed that lower glomerular filtration rate was associated with higher mortality risk after cardiac surgery. However, another study found that moderate but not mild renal impairment significantly affected the survival of patients with multivessel disease who underwent percutaneous coronary intervention or coronary artery bypass grafting [43]. This study found that having a stroke before undergoing CABG increased the mortality risk after the surgery. This finding is consistent with other studies that also reported a higher risk of death associated with preoperative stroke [44,45].

The current study had some limitations that should be acknowledged. We only recruited patients who underwent surgery at a single specialized centre. This centre serves more than 1.8 million people in Hamadan province. However, to increase the generalizability of our results, a multicentre study is needed to verify our findings. The 5-year survival rate that we estimated may not reflect the true situation due to a high rate of lost-to-follow up. The patients who were lost to follow-up might have a different mortality risk than those who remained. The main reason for the high rate of lost-to-follow up was the absence of electronic medical records and the incorrect contact information in the medical files. Moreover, we only contacted the patients once during the follow-up period, and we could not access the death records of the patients who were lost to follow-up. In addition, we did not assess the effects of education, income, leisure-time physical activity, depression, and psychological distress on the mortality rate because we lacked reliable data on these variables for the patients. These factors had missing data for some participants. Therefore, we could not calculate any risk scores due to the incomplete data on some risk factors.

## 5. Conclusions

Patients who have coronary artery disease can live longer if they monitor and control some risk factors that can worsen their condition. These include obesity, high blood glucose, opium use, and kidney problems. The results of the current study suggest that taking beta-blockers can lower the risk of death after CABG surgery.

## Figures and Tables

**Table 1:** The baseline characteristics of the study population (N = 2863).

Characteristic	N	%	Mean	SD
Age group (years)			62.94	9.38
<50	238	8.31		
50–59	758	26.48		
60–69	1150	40.17		
70–79	608	21.24		
≥80	109	3.81		
Male sex	2101	73.38		
Body mass index			26.26	4.07
Normal	1159	40.48		
Overweight	1199	41.88		
Obesity	505	17.64		
Residential area				
Urban	2187	76.39		
Rural	676	23.61		
Current smoking	858	29.97		
Hypertension	1430	49.95		
Chronic obstructive pulmonary disease (COPD)	180	6.29		
Atrial fibrillation	162	5.66		
Diabetes (≥ 126 mg/dL)	549	19.20		
Kidney failure ^†^	20	0.70		
Ejection fraction			42.83	8.80
Use of beta-blocker	918	32.06		

^†^ Glomerular filtration rate < 15 mL/min/1.73 m^2^.

**Table 2:** Survival rate of the study population who underwent CABG by year of follow-up.

Follow-Up Time	At Risk	Deaths	Lost	Survival (%)	95% CI
The first 3 days after surgery	2863	22	12	99.2	98.8–99.5
The 4th day to 1 year	2829	86	900	96.2	95.4–96.9
The 2nd year	1843	26	692	94.9	93.9–95.7
The 3rd year	1125	15	589	93.6	92.4–94.6
The 4th year	521	10	434	91.8	90.1–93.2
The 5th year	77	3	74	88.2	83.2–91.8

**Table 3:** Outcome events among the study population.

Outcome	N	%
Death		
No	2701	94.34
Yes	162	5.66
Causes of death		
Heart disease	126	77.78
Stroke	13	8.02
Pelvic fracture	2	1.23
Cancer	10	6.17
COVID-19	5	3.09
Chronic obstructive pulmonary disease	2	1.23
Diabetes	2	1.23
Kidney disease	2	1.23

**Table 4:** Univariable hazard ratio (HR) for the associations of demographic and clinical characteristics with all-cause mortality rate following coronary artery bypass graft (CABG).

Characteristic	Sample	Death	HR	95% CI	*p*-Value
Age group (years)					
<50	233	5	1		
50–59	730	28	2.71	0.63, 11.65	0.179
60–69	1094	56	3.46	0.83, 14.37	0.088
70–79	555	53	7.45	1.80, 30.84	0.006
≥80	89	20	18.04	4.13, 78.92	0.001
Sex					
Male	1987	114	1		
Female	714	48	0.92	0.60, 1.41	0.700
Residential area					
Urban	2075	112	1		
Rural	626	50	1.89	1.24, 2.67	0.002
Body mass index					
Normal	1092	67	1		
Overweight	1141	58	0.97	0.64, 1.48	0.900
Obese	468	37	1.29	0.78, 2.11	0.319
Smoking status					
Never or past	1889	116	1		
Current	812	46	1.17	0.79, 1.73	0.429
Opium use					
No	1933	112	1		
Yes	768	50	1.56	1.06, 2.28	0.023
Ejection fraction					
≥50%	1531	47	1		
<50%	1170	115	1.82	1.21, 2.73	0.004
Hypertension					
No	1362	71	1		
Yes	1339	91	1.05	0.73, 1.52	0.784
Percutaneous coronary intervention					
No	2582	155	1		
Yes	119	7	0.77	0.28, 2.09	0.607
Myocardial infarction					
No	2277	129	1		
Yes	424	33	1.11	0.69, 1.77	0.666
Coronary artery disease					
1 or 2 vessels	745	34	1		
3 or more vessels	1956	128	1.45	0.92, 2.30	0.109
Post-CABG arrhythmia					
No	2649	150	1		
Yes	52	12	0.90	0.22, 3.60	0.870
Pre-CABG arrhythmia					
No	2454	134	1		
Yes	247	28	1.68	0.99, 2.86	0.055
Use of beta-blocker					
No	1829	116	1		
Yes	872	46	0.54	0.35, 0.83	0.005
History of stroke					
No	2627	146	1		
Yes	74	16	4.02	2.29, 7.04	0.001
Cardiopulmonary bypass					
On-Pump	2152	126	1		
Off-Pump	549	36	1.52	0.99, 2.33	0.056
Chronic obstructive pulmonary disease (COPD)					
No	2532	151	1		
Yes	169	11	0.89	0.41, 1.91	0.765
Readmission times					
0	2352	115	1		
1	268	32	2.60	1.65, 4.09	0.001
≤2	81	15	2.88	1.49, 5.58	0.002
Atrial fibrillation					
No	2649	52	1		
Yes	150	12	0.98	0.22, 3.60	0.870

**Table 5:** Univariable hazard ratio (HR) for the associations of laboratory characteristics with all-cause mortality rate following CABG.

Characteristic	Sample	Death	HR	95% CI	*p*-Value
Prediabetes (blood glucose 100–125 mg/dL)					
No	2306	130	1		
Yes	391	32	1.56	0.99, 2.47	0.057
Diabetes (blood glucose ≥ 126 mg/dL)					
No	2190	120	1		
Yes	507	42	1.03	0.64, 1.66	0.888
White blood cell (×1000/mm^3^)					
Normal (≤10)	2166	93	1		
Abnormal (>10)	535	69	1.95	1.32, 2.88	0.001
Neutrophil/lymphocyte ratio					
Low (<2.15)	932	28	1		
High (≥2.15)	1769	134	1.71	1.11, 2.65	0.014
Red blood cell (Mill/mm^3^)					
Low (<3.9)	1713	87	1		
Normal (≥3.9)	988	75	1.88	1.30, 2.73	0.001
Hemoglobin (g/dL)					
Low (<11.5)	1789	90	1		
Normal (≥11.5)	912	72	1.77	1.22, 2.56	0.003
Hematocrit (%)					
Low (<36)	2026	96	1		
Normal (≥36)	675	66	2.28	1.57, 3.31	0.001
Cholesterol (mg/dL)					
Normal (<200)	918	63	1		
Borderline (200–239)	205	14	0.62	0.26, 1.46	0.275
High (≥240)	85	7	1.15	0.78, 3.87	0.177
Triglyceride (mg/dL)					
Normal (<200)	908	73	1		
Borderline (200–399)	277	7	0.35	0.14, 0.90	0.029
High (≥400)	27	5	1.95	0.85, 6.61	0.097
High-density lipoprotein (mg/dL)					
Low (<30)	150	8	1		
Normal (≥30)	1150	76	0.78	0.33, 1.84	0.570
Low-density lipoprotein (mg/dL)					
Low (<70)	303	18	1		
Normal (70–149)	879	56	1.00	0.54, 1.85	0.996
High (≥150)	118	11	1.44	0.55, 3.77	0.455
Blood urea nitrogen (mg/dL)					
Normal (<25)	2460	103	1		
High (≥25)	241	59	3.56	2.34, 5.41	0.001
Creatinine (mg/dL)					
Normal (Male: <1.4, Female: <1.1)	2386	97	1		
High (Male: ≥1.4, Female: ≥1.1	315	65	3.56	2.34, 5.41	0.001
Kidney function					
Normal (GFR 90 mL/min/1.73 m^2^)	627	16	1		
Mildly decreased (GFR 60–89 mL/min/1.73 m^2^)	1213	45	1.37	0.72, 2.64	0.339
Mildly to moderately decreased (GFR 45–59 mL/min/1.73 m^2^)	592	32	1.82	0.92, 3.61	0.087
Moderately to severely decreased (GFR 30–44 mL/min/1.73 m^2^)	223	41	4.62	2.31, 9.23	0.001
Severely decreased (GFR <29 mL/min/1.73 m^2^)	37	17	13.85	6.31, 30.39	0.001

**Table 6:** Multivariable hazard ratio (HR) for the associations of clinical and paraclinical characteristics with all-cause mortality rate following CABG.

Characteristic	HR ^†^	95% CI	*p*
Age (1-year increase)	1.05	1.02, 1.08	0.001
Body mass index (Ref: normal)			
Overweight	1.43	0.91, 2.24	0.123
Obese	2.16	1.25, 3.72	0.006
Ejection Fraction (Ref: ≥50%)			
<50%	1.61	1.06, 2.44	0.026
History of stroke	2.91	1.63, 5.21	0.001
Residential area (Ref: urban)			
Rural	1.58	1.06, 2.34	0.023
Opium use	2.08	1.40, 3.09	0.001
Use of beta-blocker	0.59	0.38, 0.91	0.018
Readmission times (Ref: 0)			
1	2.28	1.43, 3.62	0.001
>2	2.63	1.35, 5.12	0.004
Prediabetes	1.70	1.05, 2.75	0.029
Diabetes	1.07	0.65, 1.77	0.783
Kidney function			
Normal (GFR 90 mL/min/1.73 m^2^)	1		
Mildly decreased (GFR 60–89 mL/min/1.73 m^2^)	1.09	0.55, 2.18	0.803
Mildly to moderately decreased (GFR 45–59 mL/min/1.73 m^2^)	1.17	0.54, 2.55	0.693
Moderately to severely decreased (GFR 30–44 mL/min/1.73 m^2^)	2.32	0.99, 5.42	0.051
Severely decreased (GFR <29 mL/min/1.73 m^2^)	8.68	3.44, 21.91	0.001

^†^ Adjusted for all variables in the model; GFR: glomerular filtration rate.

## Data Availability

Data are contained within the article.
